# Low‐dose nivolumab for extranodal natural killer/T‐cell lymphoma, nasal type

**DOI:** 10.1002/jha2.1059

**Published:** 2024-11-29

**Authors:** Satish Maharaj, Simone Chang

**Affiliations:** ^1^ Department of Hematology/Oncology University of South Florida Tampa Florida USA

**Keywords:** immunotherapy, lymphoma, NK/T-cell, reaction

1

Natural killer (NK)/T‐cell lymphoma is a rare subtype of non‐Hodgkin lymphoma that is associated with poor outcomes. Efforts to improve treatment are urgently needed and immune checkpoint inhibitor therapies have shown promise in a series of relapsed/refractory diseases. A 59‐year‐old Hispanic male presented with near‐total obstruction of his left nasal airway. Computed tomography imaging showed left greater than right sinusitis with near‐total airway obstruction on the left side. Magnetic resonance imaging (MRI) and positron emission tomography (PET) scan showed a hypermetabolic primary mass in the left nasal cavity measuring 3.5 cm in length by 1.7 cm in width, SUV max 10.99. Nasal endoscopy showed a highly vascular exophytic tumor filling the nasal airway and sinuses; debulking and surgical biopsy were performed.

Pathology from this showed extranodal NK/T‐cell lymphoma, nasal‐type (ENKTL). Tumor cells were positive for LCA, CD2, CD3 and CD56 immunostains; negative for CD5, CD4, CD8, CD10, CD20, MUM1, CD21, BCL2, BCL6, CD79A, Cyclin D1, CD30, ALK1, Pan‐keratin, HV8, CD57, and c‐Myc immunostains. Karyotyping was normal, 46, XY[20], and bone marrow biopsy showed normocellular bone marrow without involvement and active maturing trilinear hematopoiesis. Flow cytometry analysis of the nasal mass demonstrated relatively increased natural killer cells, 66% of lymphocytes CD3‐/CD56+ with normal expression of CD16 and no immunophenotypic aberrancies without diminished or loss of CD2, CD7, and/or CD57, or abnormal uniform expression of CD8. Epstein‐Barr virus (EBV) was positive by RNA in situ hybridization (EBER Positive). Peripheral blood also was positive with EBV DNA Quantitative polymerase chain reaction level 705 copies/mL.

Using these evaluations, the patient was determined to have Stage II disease using the Chinese Southwest Oncology Group and Asia Lymphoma Study Group (CA) staging system (CASS) established in 2020 [1]. CASS Stage II is defined as a primary tumor localized to the nasal cavity or nasopharynx involving local structures, without regional lymph node involvement. Research has shown that the Ann Arbor staging system (AASS), established for Hodgkin Lymphoma, has limited predictive ability and poor prognostication for ENKTL with CASS better in discriminating survival than AASS [2, 3]. Unlike most other lymphomas, ENKTL is primarily extranodal, and therefore using the AASS, the majority of ENKTL (70%–90%) appears early (stage I/II) leading to unbalanced distribution and poor predictive accuracy.

Another model developed for patients treated with non‐anthracycline‐containing regimens is the prognostic index for NK/T‐cell lymphomas (PINK‐E) (negative scoring parameters: age  >60 years, stage III/IV disease, distant lymph node involvement, non‐nasal subtype, and detectable presentation EBV DNA) [4]. Using PINK‐E, this patient would be classified as low‐risk. Using the PINK‐E model, differences between low‐risk and high‐risk groups were noted, but a significant difference was not found for overall survival and progression‐free survival between the low‐risk and the intermediate‐risk group (*p* = 0.068 and *p* = 0.079, respectively) [4].

The patient proceeded to upfront treatment using the mSMILE approach as previously described, involving modification with dexamethasone, methotrexate, ifosfamide, L‐asparaginase, and etoposide for two cycles followed by Intensity‐Modulated Radiotherapy (IMRT) [5]. Given the aggressive disease and delayed presentation, cycle 1 was administered urgently, and supportive medications were used, including carnitine and ursodiol. He tolerated two cycles of mSMILE without any dose‐limiting toxicity and following induction proceeded to 30 fractions of definitive IMRT. A post‐treatment restaging PET scan at 3 months post‐radiation showed partial response in the left nasal cavity primary with a Deauville score of 4; restaging investigations showed residual disease. EBV level retesting showed improvement from 700 to <200 copies/mL, but remained detectable in peripheral blood.

The patient discussed hematopoietic cell transplantation or further chemotherapy but declined these approaches, deciding on a further 3 months of observation. At 6 months post‐radiation he was found to have bilateral neck lymph node involvement. Serum EBV remained detectable and circulating tumor DNA (ctdNA) using the tumor‐informed Signatera assay was also positive (Figure 1). After a discussion on treatment options, the patient opted to proceed with nivolumab using a standard dose of 240 mg intravenously, planned every 2 weeks. With cycle 1 he experienced severe back pain and flushing during the latter half of the infusion consistent with a drug‐induced reaction. This was managed supportively and he was rechallenged with cycle 2, using dexamethasone and diphenhydramine premedication, however with the standard dose he again experienced infusion reaction in the latter half of the infusion with severe back pain and hypotension.

**FIGURE 1:**
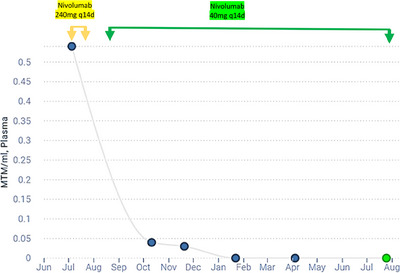
Response in circulating tumor DNA with low‐dose nivolumab monotherapy.

Given these reactions, he decided to trial low‐dose nivolumab at 40 mg every 2 weeks dosing. This approach was extrapolated from the reported series of three patients in Hong Kong [2]. The patient tolerated this low‐dose approach well and had clinical, radiographic, and ctDNA resolution of the disease (Figure 1). At 1 year of therapy, the patient continues in remission, now 18 months post‐radiation. This case adds to the three previously reported, showing the efficacy of low‐dose nivolumab in the treatment of extranodal NK/T‐cell lymphoma, with longer follow‐up than the previously reported case reports.

Low‐dose nivolumab has shown efficacy in acute lymphoblastic leukemia relapse after allogeneic transplantation, refractory classical Hodgkin lymphoma, and advanced head and neck squamous cell carcinoma [6, 7, 8, 9]. While in the current case, low‐dose therapy was used out of necessity, accumulating evidence suggests that the dose–response correlation with immunotherapy is quite distinct from chemotherapy, and immunotherapy could be administered at doses considerably lower than those currently used without compromising efficacy [10]. NK/T‐cell lymphoma is an aggressive disease with high mortality and incorporating low‐dose immunotherapy in consolidation seems promising, albeit from a limited number of cases. Further studies are needed to validate this approach compared to standard dose immunotherapy.

## AUTHOR CONTRIBUTIONS

The manuscript has been read and approved by all the authors, the requirements for authorship have been met, and each author believes that the manuscript represents honest work.

## CONFLICT OF INTEREST STATEMENT

The authors declare no conflict of interest.

## FUNDING INFORMATION

The author received no specific funding for this work

## ETHICS STATEMENT

The authors have confirmed ethical approval statement is not needed for this submission.

## PATIENT CONSENT STATEMENT

The authors have confirmed patient consent statement is not needed for this submission.

## CLINICAL TRIAL REGISTRATION

The authors have confirmed clinical trial registration is not needed for this submission.

## Data Availability

The authors confirm that the data supporting the findings of this study are available within the article and its Supporting Information materials.
